# Effects of histatin-1 peptide on human corneal epithelial cells

**DOI:** 10.1371/journal.pone.0178030

**Published:** 2017-05-23

**Authors:** Dhara Shah, Marwan Ali, Deepak Shukla, Sandeep Jain, Vinay Kumar Aakalu

**Affiliations:** 1 Lacrimal Cell Biology Laboratory, University of Illinois at Chicago, Department of Ophthalmology and Visual Sciences, Chicago, United States of America; 2 University of Illinois at Chicago, Department of Ophthalmology and Visual Sciences, Chicago, United States of America; Cedars-Sinai Medical Center, UNITED STATES

## Abstract

**Purpose:**

Ocular surface and corneal epithelial wounds are common and potentially debilitating problems. Ideal treatments for these injuries would promote epithelial healing without inflammation, infection and scarring. In addition the best treatments would be cost-efficient, effective, non-toxic and easily applied. Histatin-1 peptides have been shown to be safe and effective enhancers of epithelial wound healing in other model systems. We sought to determine whether histatin-1 peptides could enhance human corneal epithelial wound healing *in vitro*.

**Methods:**

Histatin-1 peptides were applied to human corneal epithelial cells and compared over useful dose ranges in scratch assays using time-lapse microscopy. In addition, path finding analysis, cell spreading assays, toxicity and proliferation assays were performed to further characterize the effects of histatin-1 peptide on human corneal limbal epithelial (HCLE).

**Results:**

Histatin-1 enhanced human corneal epithelial wound healing in typical wound healing models. There was minimal toxicity and no significant enhancement of proliferation of corneal epithelium in response to histatin-1 application. Corneal epithelial spreading and pathfinding appeared to be enhanced by the application of histatin-1 peptides.

**Conclusions:**

Histatin -1 peptide may enhance migration of HCLE cells and wound healing *in vitro*. These peptides may have benefit in corneal epithelial wounds and need to be investigated further.

## Introduction

The outer layer of the cornea, the corneal epithelium, serves as a physical barrier against the environment and as a line of defense to prevent infectious and/or toxic agents from penetrating the eye [1]. Ocular surface injuries and burns may cause significant loss of function and decrement in quality of life [2]. The corneal epithelium is an integral part of the wound healing process, in the event of injury to the cornea.

Wound healing in the corneal epithelium is a multi-phased, complex phenomenon that involves cell migration, proliferation, epithelial-mesenchymal cross-talk, various growth-factors and extracellular matrix changes [3–5]. Enhancement of corneal epithelial wound healing is desirable to prevent infection, scarring, neovascularization and visual loss in the context of injury. A topically applied treatment which promotes corneal epithelial migration prevents infection, and decreases the chances of scarring would be helpful in the management of corneal epithelial wounds.

Non-healing or persistent corneal epithelial wounds can lead to stromal perforation, intraocular infection, intraocular tissue and fluid loss, pain and loss of vision [6]. Numerous conditions can lead to non-healing corneal wounds, including herpes simplex, herpes zoster, exposure keratopathy, severe dry eye, diabetic keratopathy, diabetic vitrectomy, neurotrophic keratopathy, corneal transplantation, corneal burns and limbal stem cell deficiency. Treatments for non-healing corneal wounds are limited with no specific therapy available [3]. It has been noted that epithelial migration is a critical aspect in the healing of persistent corneal epithelial defects [6].

Histatins are the major wound-closing factors of human parotid saliva [7]. They are natural antimicrobial peptides that are comprising of two genes and 13 proteins, of these histatin-1,3 and 5 are the principal members of the family. Histatin-1 has been shown to have anti-bacterial, anti-fungal activities and stimulates epithelial wound healing in several tissue types and culture systems [7]. In particular, the c-terminal wound healing domain of histatin-1 has been the subject of study. The wound healing effects of histatin-1 appear to be independent of epidermal growth factor (EGF), but may also synergistically enhance recombinant human epidermal growth factor (rhEGF) effects on cell migration in some model systems [8]. Given the utility of histatin-1 in other model systems to stimulate epithelial cell migration, we investigated its ability to enhance wound-healing in cells of the corneal epithelium.

## Materials and methods

### Ethics statement

This study was completed as part of an approved Institutional Review Board (IRB) protocol and processed by The University of Illinois at Chicago (UIC). Human corneal limbal epithelial (HCLE) cells were generously provided by Sandeep Jain (University of Illinois at Chicago, Chicago, IL, USA) in a deidentified manner [9].

### Cell culture

HCLE cells were thawed from liquid nitrogen and cultured in keratinocyte-serum free medium (K-SFM, ThermoScientific, Waltham, MA, USA) supplemented with 0.2 ng/mL rhEGF (ThermoScientific, Waltham, MA, USA), bovine pituitary extract (ThermoScientific, Waltham, MA, USA) and 1% Amphotericin B (ThermoScientific, Waltham, MA, USA). Standard cell culture conditions (37°C, 5% CO_2_, >95% humidity) were used during routine passages, as has been done previously [10]. The culture medium was replaced every 48 hours after seeding.

### Peptide synthesis and purification

Linear histatin-1 peptide was synthesized using the stepwise solid-phase method by the 9-fluorenylmethoxycarbonyl (Fmoc) chemistry on the Wang resin (AnaSpec, Fremont, CA, USA) with a 12 channel multiplex peptide synthesizer (Protein Technologies, Tucson, AZ, USA) according to the manufacturer’s protocol. The crude peptides were then purified on a preparative Kinetex reversed-phase C18 column, 150 x 21.1 mm (Phenomenex, CA, USA) using a BioCad Sprint (Applied Biosystems, CA, USA). The pure peptide fraction were identified by matrix-assisted laser desorption/ionization time-of-flight mass spectrometry (MALDI-TOF MS) or electrospray ionization mass spectrometry (ESI MS) and lyophilized as appropriate. 1000μM and 500μM stock solution of histatin-1 was made by dissolving lyophilized peptide in phosphate buffer saline (PBS) and utilized in all the studies described below.

### *In vitro* scratch assay

HCLE cells were cultured in a 24 well plate at 5x10^4^ seeding density, and allowed to incubate for 24-hour period in K-SFM media to form a confluent monolayer. Subsequently a straight line scratch mark was made with sterile P200 pipette tip. The cells were then washed with media to remove cellular debris. Wounded areas were then treated with histatin-1 at 5, 10 and 50 μM concentrations in complete K-SFM medium. Scratches were photographed microscopically (Zeiss Cell Observer SD live cell Imaging system, CA, USA) at 0, 3, 5, 7, 9 and 11 hours. Control cells were exposed to “vehicle only” (PBS). The cell free area at each time-point was measured using Image J software (Image J 1.47v, NIH, Thornwood, Bethesda, MD, USA) and only closely matching areas were selected for analysis. To ensure that the similar wound areas were compared, the produced wound area was traced and measured for three positions within each well. The average of these three positions at different time-point was used to calculate percentage closure. Percentage closure was calculated by dividing total area of the wound at different time points by that of the total area of the initial wound. Pathfinding was assessed using the Image J plug-in “M-Track J” by following the path of a single cell (10 cells per wound) from wounding to closure. Length of the path from one side of the wound was divided by half the linear width of the wound in order to give a ratio that represents the non-random/efficiency of epithelial migration across the cell free area of the scratch. Distance travelled was measured in micrometers.

### Immunofluorescence imaging/ cell spreading assay

The cell spreading assay was performed as described in the literature [10]. Briefly, HCLE cells were seeded in low density to visualize single cell populations in K-SFM medium. The cells were then treated with histatin-1 at 5, 10, 50 μM and “vehicle only” (PBS) controls for 24 hours at 37°C in 5% CO_2_, >95% humidity. For Phalloidin staining, cells were fixed in 4% Paraformaldehyde, permeabilized with PBS containing 0.1% Triton X-100. Followed by incubation of cells with Oregon green 488 Phalloidin (Thermo Fisher, Waltham, MA, USA) for 30 minutes at room temperature. Thereafter the mounting medium with 4′,6-diamidino-2-phenylindole (DAPI) was applied before covering the chamber slide with glass coverslips. Images of the stained HCLE cells were captured and analyzed using the Zeiss LSM 710 Confocal Microscope. The area of the individual cells (n = 60 “vehicle only” (PBS) control n = 60 for 5μM, n = 62 for 10 μM, n = 60 for 50 μM for treatment) on phase contrast image was calculated using Image J software (Image J 1.47v, NIH, Thornwood, Bethesda, MD, USA). Measurements were taken by an observer masked to treatment status.

### Cell proliferation and toxicity assays

#### MTT

The 3-(4,5-Dimethylthiazol-2-yl)-2,5-Diphenyltetrazolium Bromide (MTT) assay was performed on HCLE cells. The cells were cultured on 96-well cell plate at 1 x 10^4^ cells/well seeding density in K-SFM medium and were treated with histatin-1 samples at 0.5, 1, 5, 10, 50, 100, 200, 400 μM and “vehicle only” (PBS) control for 24 hours at 37°C in 5% CO_2_, >95% humidity. After 24 hours, MTT dye solution (Promega, Madison, WI, USA) was added to the cells. After 4 hours of incubation at 37°C in 5% CO_2_, >95% humidity, stop solution was added and the developed color was read using a microplate reader at 570nm (SynergyH1, BioTek, Winooski, VT, USA). A no cell blank was used to subtract background absorbance from the original values. This experiment was performed in triplicate. The data were normalized to “vehicle only” (PBS) control.

#### LDH

Toxicity of histatin-1 was evaluated by measuring lactate dehydrogenase (LDH) activity released in the media during the exposure to peptides. Histatin-1 exposure was measured using the CytoTox 96^®^ nonradioactive assay (Promega, Madison, WI, USA) following the manufacturer instructions. The HCLE cells were cultured on 96-well plate at 1 x 10^4^ cells/well seeding density in K-SFM medium and were treated with histatin-1 samples at 0.5,1, 5, 10, 50, 100, 200, 400 μM and “vehicle only” (PBS) control for 24 hours at 37°C in 5% CO_2,_ >95% humidity. For maximum LDH release control, HCLE cells were lysed using 1X lysis solution (“100% lysis control”) for 45 minutes prior to adding CytoTox 96 reagent. After the lysis the CytoTox 96 reagent was added to the “vehicle only” (PBS) control, histatin-1 treated samples and complete LDH release control were incubated for 30 minutes at room temperature. After 30 minutes, stop solution was added and the developed color was read using a microplate reader at 490nm (SynergyH1, BioTek, Winooski, VT, USA). A no cell blank was used to subtract background absorbance from the original values. This experiment was performed in triplicate. The data were normalized to “vehicle only” (PBS) control.

#### BRDU

Cell proliferation analysis was performed using cell proliferation Bromodeoxyuridine (BrdU) incorporation assay (Roche, Indianapolis, IN, USA) according to manufacturer’s protocol. HCLE cells were cultured on a 96 well plate at 1 x 10^4^ cells/well seeding density in K-SFM medium and were treated with histatin-1 samples at 0.5, 1, 5, 10, 50, 100, 200, 400 μM and vehicle only (PBS) control for 24 hours at 37°C in 5% CO_2_, >95% humidity. Thereafter the BrdU labeling reagent was added to the histaitn-1 treated and “vehicle only” (PBS) control samples and incubated for 2 hours at 37°C in 5% CO_2_, >95% humidity. Cells were fixed using FixDenat solution for 30 minutes at room temperature. This was followed by incubation of cells with Anti-BrdU-POD solution for 90 minutes at room temperature thereafter cells were washed three times using 1X washing solution. Substrate solution was added to the cells and plate was read using luminometer (SynergyH1, BioTek, Winooski, VT, USA) within 10 minutes of adding substrate solution. For background control, BrdU labeling solution was eliminated. Subsequently the background values were subtracted from the original values to obtain true BrdU uptake results. This experiment was performed in triplicate. The data was normalized to “vehicle only” (PBS) control.

### Statistical analysis

Statistical software (GraphPad Prism 6; GraphPad Software, Inc., La Jolla, CA, USA) was used to analyze the data. Data represents the means of triplicates used in all the experiments and the error bars represent standard error of the mean (SEM). P values were calculated using 2-way ANOVA with Bonferroni’s posttest in the wound healing assay. Student’s t-test (Unpaired, two tailed) was used to calculate P values in pathfinding experiment. One-way ANOVA with Bonferroni’s multiple comparison test was used for pathfinding assays, MTT assays and LDH assays and BrdU Assays. Asterisks in wound healing assays denote significant difference: ***P ≤ 0.001, asterisks in path finding assay denote significant difference: ** *P* ≤ 0.01, asterisks in cell spreading, MTT assay, BrdU and LDH assay denote significant difference: *P ≤ 0.05, **P ≤ 0.01 and ***P ≤ 0.0001.

## Results

**Fig 1:**
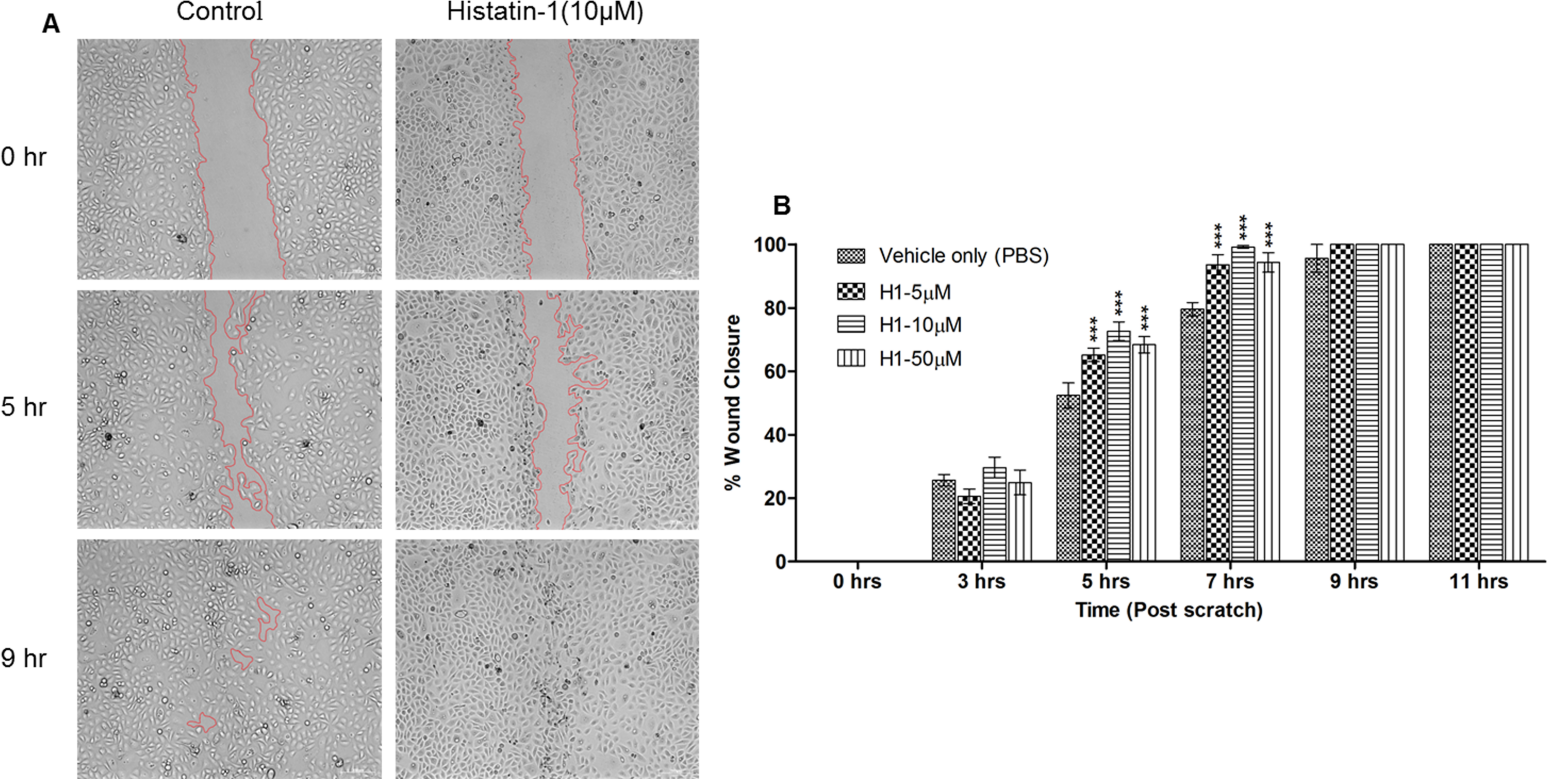
Histatin-1 improves rates of wound closure in an *in vitro* scratch assay. (A) Representative images from wound healing assay of HCLE cell cultures treated with histatin-1 demonstrating enhancement of wound closure compared to “vehicle only” (PBS) control; scale bar = 200μM (only 10 μM shown) (B) Summary bar graph illustrating percentage wound closure at indicated time points during the scratch assay. Notable is the statistically significant improvement in wound closure times. Each condition was compared to “vehicle only” (PBS) control. P values: ***P ≤ 0.001 as determined by two-way ANOVA with Bonferroni’s posttest. The error bar represents means ± SEM of three scratched areas imaged per condition.

We assessed the effects of histatin-1 application on corneal epithelial wound healing using time lapse microscopy and a standard scratch assay. Fig 1 demonstrates the effects of application of soluble histatin-1 at different concentrations to HCLE cells in culture after wounding. Histatin-1 exposed wounds closed more quickly than did control cells. The effect of histatin-1 application in accelerating wound healing was statistically significant and wounds with treated cells closed sooner than cells exposed to “vehicle only” (PBS) control.

**Fig 2:**
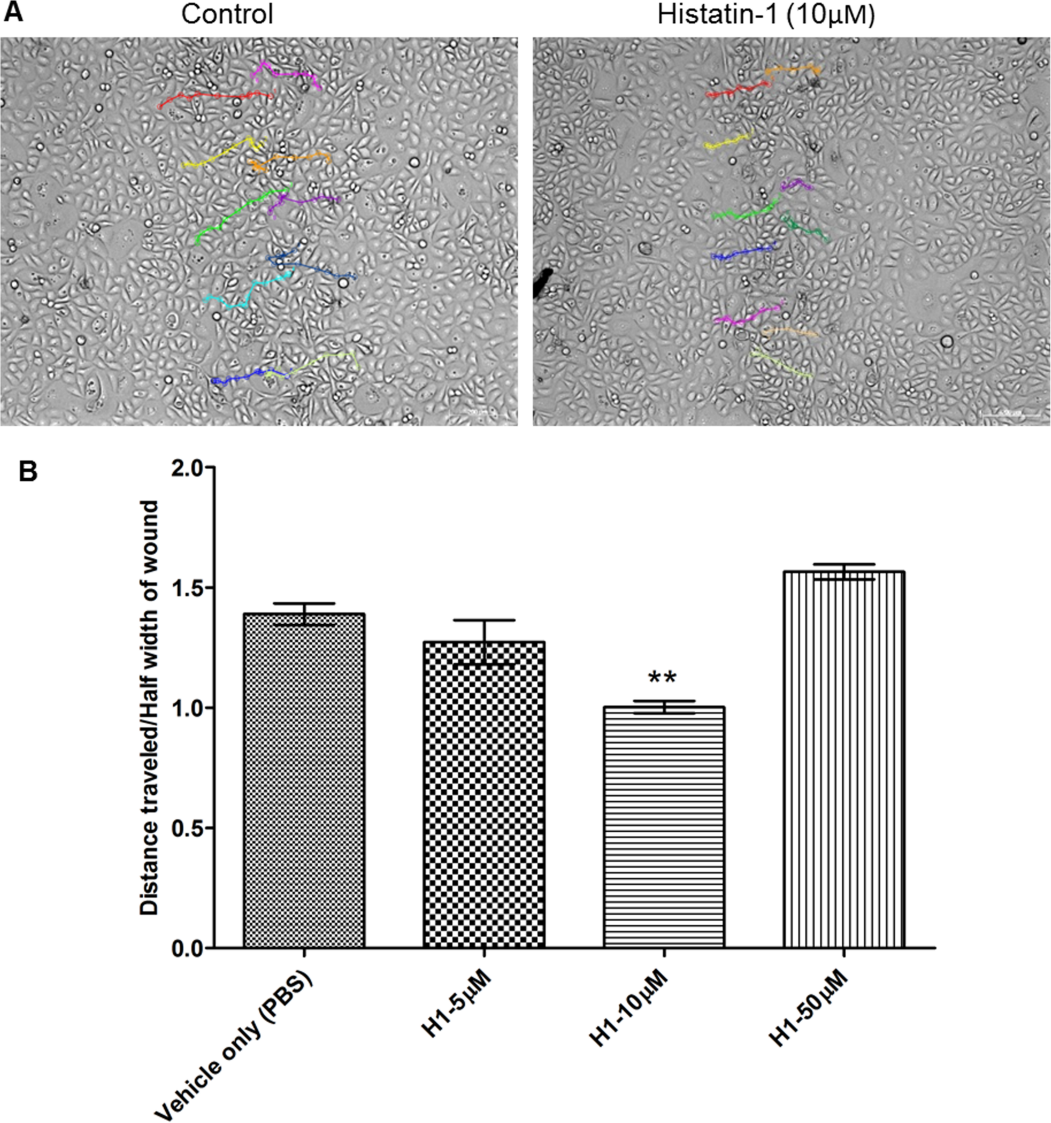
Histatin-1 enhances pathfinding of HCLE cells across a scratch wound *in vitro*. (A) Migratory paths of individual cells along the edge of the wound, showing shorter path length with histatin-1 vs. “vehicle only” (PBS) control; scale bar = 200μM (only 10 μM shown) (B) Histatin-1 reduces path length to wound closure in comparison to “vehicle only” (PBS) control. Each condition was compared to “vehicle only” (PBS) control. Length of the path from one side of the wound was divided by half the linear width of the wound in order to give a ratio that represents the non-random/efficiency of epithelial migration across the cell free area of the scratch. P values: ** *P* ≤ 0.01using one-way ANOVA with Bonferrroni’s multiple comparison test. The error bar represents means ± SEM of three scratched areas imaged per condition.

In order to better understand the improvement in corneal epithelial wound closure we investigated epithelial pathfinding in scratch assays. Fig 2 demonstrates the changes in pathfinding of HCLE cells across the wound defect with or without histatin-1 application. We found that there was a significantly shorter path for cells across the wound with application of histatin-1 at 10 μM as compared with “vehicle only” (PBS) control. This difference was noted to be statistically significant. We noted a small, but statistically insignificant increase in path length when using 50 μM of histain-1.

**Fig 3:**
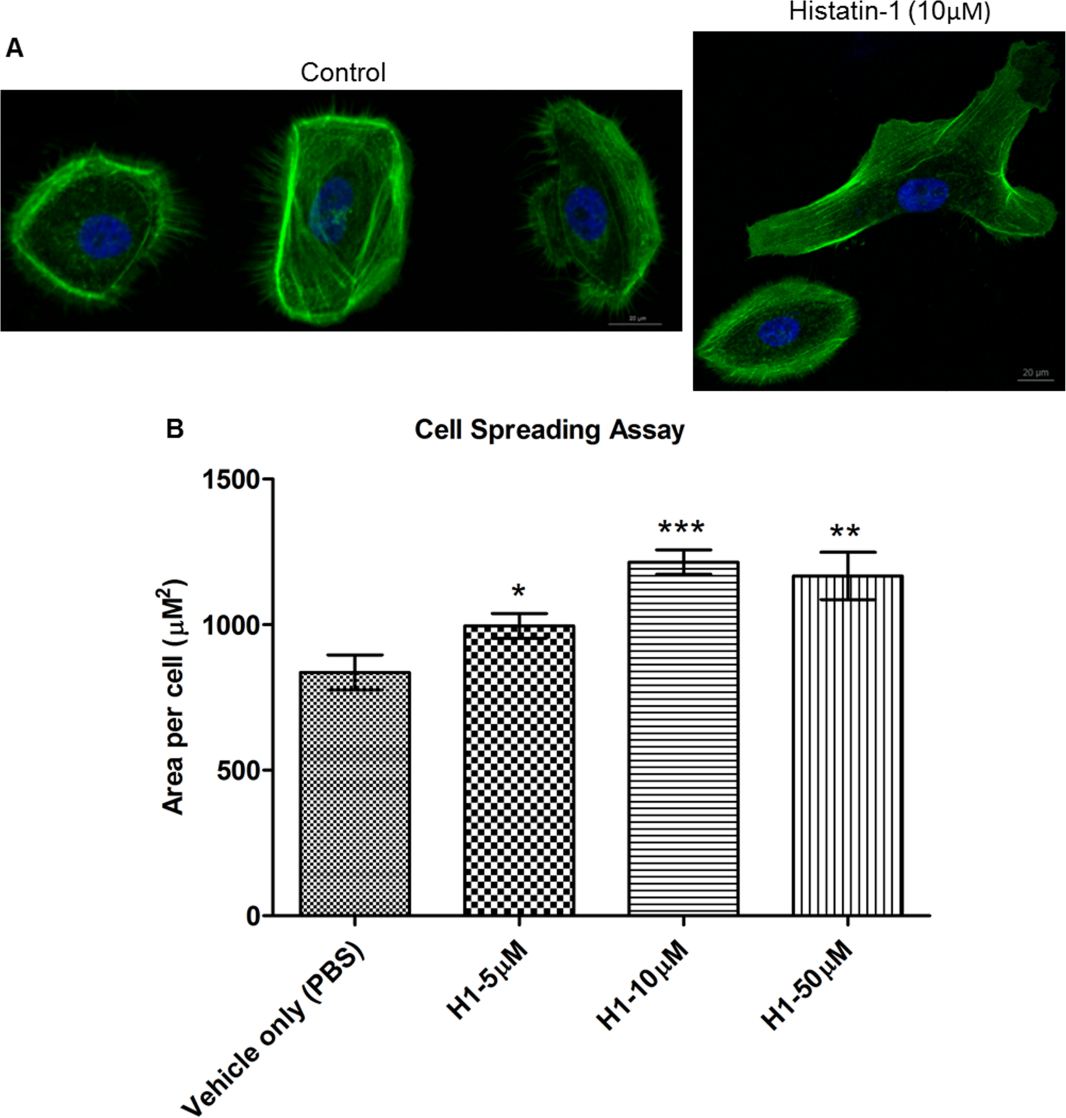
Histatin-1 enhances cell spreading. Cell spreading assay using cell surface area measurements and visualization of actin in HCLE cells via phalloidin staining. Noted is the statistically significant increase in cell surface area after application of histatin-1 compared with “vehicle only” (PBS) control. (A) Representative images of HCLE cell spreading 24 hours after seeding in the presence or absence (“vehicle only” (PBS) Control) of histatin-1. Green, actin; blue, nuclei. Scale bar = 20μM. (only 10 μM shown) (B) Average surface area per cell was quantified from images similar to those in A. Measurement of average individual cell surface area is significantly higher with all tested concentrations of histatin-1 treatment. Each condition was compared to “vehicle only” (PBS) control. P values: * *P* ≤ 0.05, ** *P* ≤ 0.01, *** *P* ≤ 0.0001 using student’s T test. The error bar represents means ± SEM; (n = 60 “vehicle only” (PBS) control n = 60 for 5μM, n = 62 for 10 μM, n = 60 for 50 μM of histatin-1 treatment).

We then sought to determine if cell spreading was enhanced in HCLE cells after application of histatin-1 peptide at different concentrations. Cell spreading was determined using multiple measurements of individual cells in low-density culture after histatin-1 application and compared with “vehicle only” (PBS) control. In addition, visualization of the cell actin skeleton was performed using phalloidin staining. Fig 3 demonstrates statistically significant increase in cell surface area after application of histatin-1 compared with control in all tested concentrations, but a peak effect with 10 μM of the peptide.

**Fig 4:**
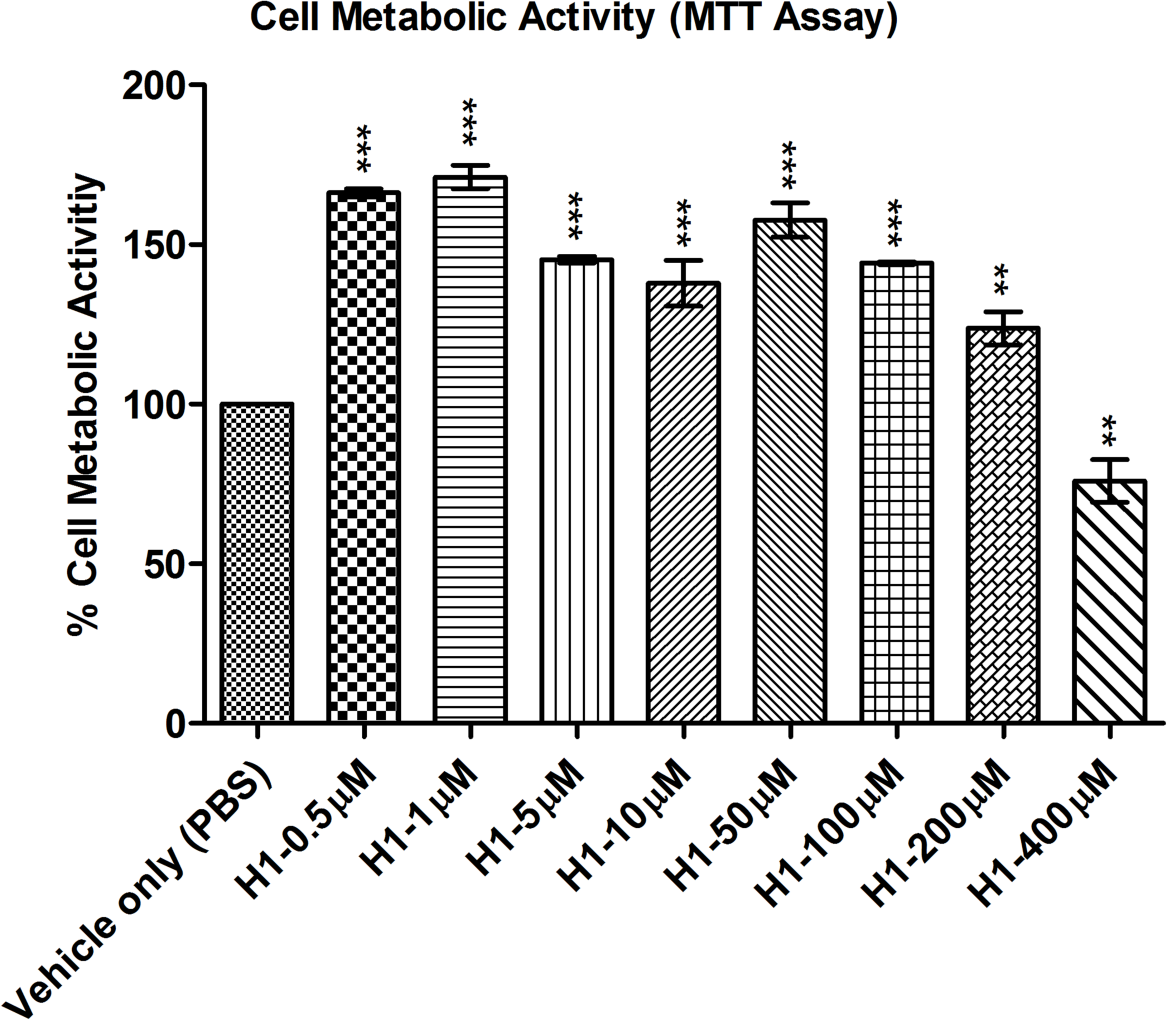
MTT assay for cell metabolic activity. Noted is significant increase in cell metabolic activity with histatin-1 application compared with “vehicle only” (PBS) control upto concentrations of 200 μM, and a significant decrease in metabolic activity at 400 μM. Each condition was compared to “vehicle only” (PBS) control. P values: ** *P* ≤ 0.01, *** *P* ≤ 0.0001 using one-way ANOVA with Bonferrroni’s multiple comparison test. The error bar represents means ± SEM of the triplicate measurement.

**Fig 5:**
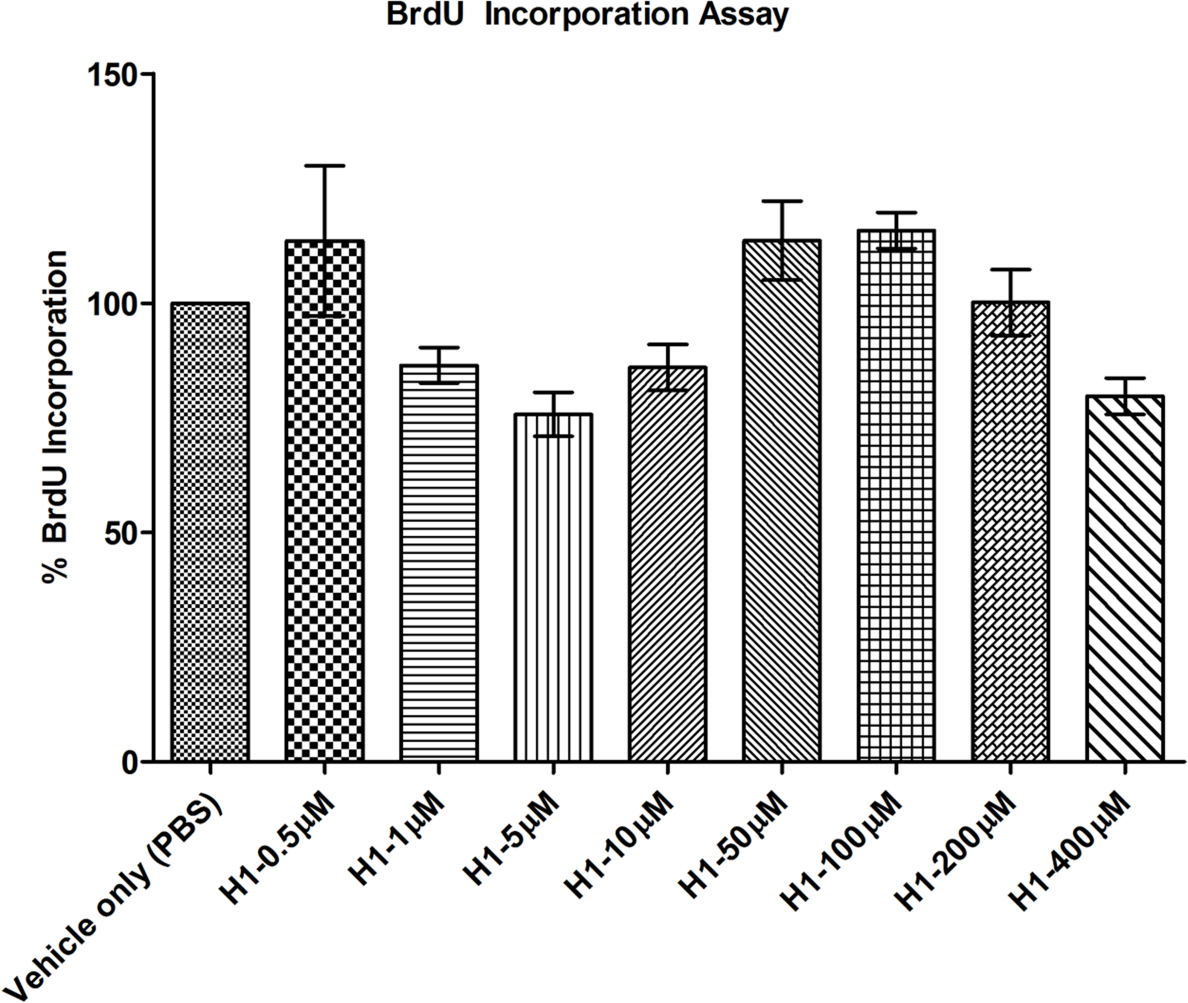
BrdU assay for cell proliferation. Noted is no statistically significant increase in cell proliferation in the BrdU incorporation assay at any tested concentraton. Each condition was compared to “vehicle only” (PBS) control. The error bar represents means ± SEM of the triplicate measurement.

**Fig 6:**
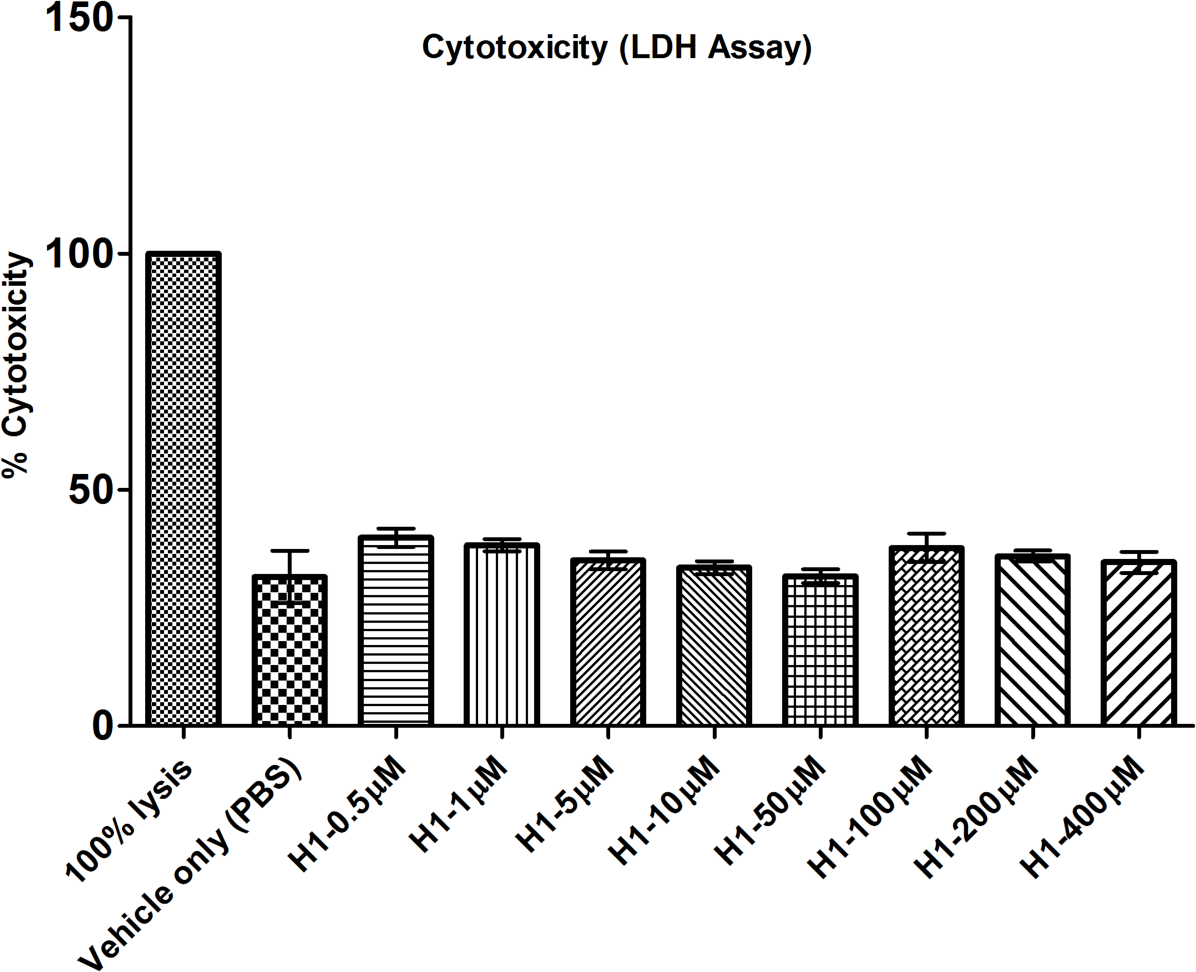
LDH assay for cytotoxicity. Demonstrates a lack of significant toxicity to HCLE cells after application of histatin-1 compared with “vehicle only” (PBS) control. Each condition was compared to “vehicle only” (PBS) control. There were no statistically significant differences noted between “vehicle only” (PBS) control and the histatin peptides. The error bar represents means ± SEM of the triplicate measurement. 100% lysis refers to the manufacture’s supplied lysis buffer, causing 100% cell death.

We then performed a series of tests to assess for cell metabolic activity (MTT) (Fig 4), proliferation (BrdU) (Fig 5) and toxicity (LDH) (Fig 6). These cell metabolism, proliferation and toxicity assays show effects of histatin-1 peptide on HCLE compared with “vehicle only” (PBS) control. Notably there was an increase in cell metabolism seen in MTT assays but no statistically significant increase in proliferation (BrdU) at any concentration. LDH assessment showed no increase in cell toxicity at any tested concentration of histatin versus “vehicle only” (PBS) control [11]. Taken together these results suggest that the effects histatin-1, *in vitro¸* on HCLE cells is a small increase in metabolic activity, minimal toxicity, and no significant induction of proliferation.

## Discussion

Wounding of the corneal epithelium is an extremely common, potentially painful, and sometimes blinding problem [12]. Corneal epithelial wounds heal through the complex interaction of multiple cell types, growth factors, extracellular matrix remodeling and requires the interplay of multiple signaling pathways in order to effectively protect the corneal stroma, prevent infection and maintain corneal clarity [3]. In addition, wound healing of the ocular surface poses some tissue specific considerations that are different than other areas of the body in order to maintain corneal clarity. Ideal corneal healing requires that the wound heals without significant scarring/tissue melting, vascular in-growth, infection and inflammation [3, 12].

Persistent epithelial corneal wounds are difficult problems that occur in a number of disease states. Persistence of these wounds can lead to stromal scarring, chronic inflammation, infection, loss of vision and even perforation of the eye [6]. Although proliferative capacity of the corneal epithelium in some disease states may be maintained in persistent epithelial defects, migration of corneal epithelium across a denuded corneal stroma is often impaired. It has been reported that migration of corneal epithelium is the critical aspect of healing of these defects [6]. No specific, effective treatment has been found to treat these common and debilitating wounds [3]. An ideal treatment for these defects would promote epithelial migration, mitigate inflammation, reduce potential for scarring, decrease chances of ocular infection, have limited toxicity and be easily applied.

Oral mucosal wounds are known to heal rapidly and without significant scarring. Saliva has a number of pro-wound healing properties. Thus the study of potential wound healing elements in human saliva has been the subject of numerous investigations. In addition, salivary gland transplantation has been utilized in the treatment of severe ocular surface diseases [13]. Thus, wound healing strategies from the oral mucosa may have implications in ocular surface disease.

Histatins are the primary wound healing factors in human saliva, and are likely to be more important in oral mucosal wound healing than EGF, or other factors present in saliva [7, 14]. Histatins were originally described as antibacterial histidine-rich peptides in saliva [15], but have since been noted for their anti-fungal and wound healing activities [7]. In addition, histatins appear to have a variety of anti-proteolytic abilities and have been found to inhibit matrix metalloproteinase (MMP) activity. The metal binding domains of histatin peptides may play a role in MMP inhibition. Histatins also inhibit lipopolysaccharides (LPS), which may contribute to decreased inflammation and reduced attraction of innate immune system players, which release MMPs and other proteases [14]. Although the great majority of studies on histatin peptides have been performed in the oral biology space, histatin-1 has been demonstrated to be expressed highly in human lacrimal gland and accessory lacrimal gland [15–17].

The wound healing properties of histatins are of particular interest in their potential use as therapeutics for ocular surface disease. Histatin peptides may be well suited for application in treating ocular surface wounds since they promote epithelial cell migration, spreading, decrease protease activity, demonstrate anti-fungal and anti-bacterial properties and may decrease inflammatory LPS effects. Furthermore, these peptides are easily synthesized and amphipathic- potentially allowing topical ophthalmic application [18, 19]. Taken together, these data suggest histatin peptides as a promising source of therapeutics in ocular surface wound healing and infection treatment/prevention.

The mechanism of action for histatin-1 peptide enhancement of wound healing is unknown. Previous studies in non-corneal tissues have demonstrated that histatins promote cell spreading and migration; their wound healing effects are thought to occur in an EGF independent manner without significant stimulation of epithelial proliferation [7]. Wound healing through histatin is likely mediated by g-protein coupled receptors (GPCR) through the extracellular signal–regulated kinases (ERK)1/2 mitogen-activated protein kinases (MAPK) pathways. Moreover, it has been reported that an unidentified GPCR pathway (Galpha-i linked) is critical for histatin peptide effects on cell migration [20]. Studies in other tissues on MAPK pathways have demonstrated an increase in cell-substrate and cell-cell adhesion with increased trans-epithelial resistance [21] and independence from p38 MAPK (proliferative) pathway inhibitors [7].

Our own preliminary (unpublished data) suggest that histatin peptides have effects on wound healing, GPCR and cell signaling pathways in human corneal epithelium, which is consistent with the literature. In particular, we have found gene expression changes in cell migration pathways, LPS response and GPCR signaling. At the protein level we have found initial results which suggest that histatin peptides can affect the MAPK signaling pathways in corneal epithelium, as has been noted in other tissue types [7, 11]. Further experimentation to elucidate the underlying mechanisms of action of hisatin peptides in corneal epithelia are on-going in our laboratory.

In the present study, we found that the application of histatin-1 at various, physiologic, concentrations enhances wound healing in cultured HCLE cells *in vitro*. This improvement in rates of corneal epithelial healing was demonstrated in a time-lapse imaged scratch assay over a concentration range of 5–50μM of solubilized histatin-1. [8, 22]. In addition, we assessed cell pathfinding by comparing path length traveled among the different concentrations of peptides and compared with “vehicle only” (PBS) control. We noted that there was a statistically significant, reduction in path length with addition of histatin-1 compared with control at a concentration of 10μM. We also assessed the effects of histatin-1 application to cell spreading and found a statistically significant increase in corneal epithelial cell area after application of histatin-1 versus control at all tested concentrations, with peak effect at 10μM. Taken together these data suggest that histatin-1 peptide may enhance corneal epithelial migration and spreading, with a peak effect at a concentration of 10μM. A dose dependent effect, with ideal concentrations for each effect of the peptide (migration, pathfinding etc), may explain the slightly worsened pathfinding (statistically insignificant) of HCLE at 50μM, but retained acceleration of wound healing and cell spreading. This supposition requires further analysis. Other authors have also found that 10μM histatin-1 to be the most efficacious dose for enhancing wound healing in other cell types [11]. However, our unpublished data in different cell types (SV40 immortalized human corneal epithelium), showed a consistent dose dependent increase in efficacy up to 50μM, suggesting that different cell types could exhibit variable peak effect concentrations.

With regards to the potential effects of histatin-1 peptides on cell proliferation and toxicity we found a number of interesting results. Many assays are available to evaluate cell metabolism, toxicity, proliferation, viability and death [23]. MTT assays have been utilized to assess for cell viability and toxicity [12], increases in MTT assay values can imply increased metabolic activity or proliferation. Decreases in MTT assay results can be associated with cell toxicity or decreased metabolic activity. LDH assays are a measure of cell death and toxicity [23–25]. BRDU assays are utilized to measure cell proliferation, and can be used to distinguish between increased metabolic activity and proliferation in conditions with increased MTT activity [26]. Each of these tests performed individually can provide an incomplete picture of the effects of a compound on cell death, proliferation, toxicity, metabolism and viability. When performed in concert, multiple cell viability assays can yield a strong foundation for future *in vivo* experiments [23].

In our study, MTT analyses demonstrated increased metabolic activity in HCLE cells exposed to histatin-1 in concentrations up to, but not including 400 μM. Confirmatory BrdU analysis demonstrated no significant increase in cell proliferation at any concentration. At greater than 200μM concentrations there was a statistically significant decrease in metabolic activity in HCLE cells using the MTT assay but this did not meet the established threshold of 60% viability in MTT assays to predict significant ocular toxicity [12]. Additionally, confirmatory toxicity evaluation via LDH assay analysis, did not demonstrate any significant toxicity in all tested concentrations of histatin-1 up to 400μM (as compared with “vehicle only” (PBS) control. These results correlate well with other studies, in multiple tissue types suggesting that histatin-1 does not cause a strong proliferative effect on corneal epithelia [7, 27]. Based on these *in vitro* results, we believe that histatin peptides are non-toxic to HCLE cells and do not induce significant proliferation in these cells. Future *in vivo* analyses will need to be performed to confirm these data.

## Conclusions

Thus, histatin-1 enhances corneal epithelial wound healing and may enhance corneal epithelial migration and path finding *in vitro*. Limitations of this study include the *in vitro* nature of the studies and the use of an immortalized cell line. In addition, it should be noted that wound healing is a complex process that is tissue and cell type dependent. It involves numerous interactions among different cell types, local and distant factors and is difficult to model completely *in vitro*. We believe these limitations are mitigated somewhat by the well vetted nature of the cell line, complimentary nature of the different experimental assays employed, the standardization of the methodology and the statistically significant differences found in the study. Nevertheless, *in vitro* experiments such as this one support future experiments and allow for screening of potentially useful compounds to advance the development of therapeutics [27]. Future mechanistic studies and studies using *in vivo* models will be necessary to make stronger conclusions regarding the applicability of histatin peptides in the enhancement of corneal epithelial migration and/or wound healing.

## References

[1] Leong YY, Tong L. Barrier function in the ocular surface: from conventional paradigms to new opportunities. Ocul Surf 2015 4;13(2):103–109. doi: 10.1016/j.jtos.2014.10.003 25881994 10.1016/j.jtos.2014.10.003

[2] Singh P, Tyagi M, Kumar Y, Gupta KK, Sharma PD. Ocular chemical injuries and their management. Oman J Ophthalmol. 2013 May-Aug;6(2):83–86. doi: 10.4103/0974-620X.116624 24082664 10.4103/0974-620X.116624 PMC3779420

[3] Wirostko B, Rafii M, Sullivan DA, Morelli J, Ding J. Novel Therapy to Treat Corneal Epithelial Defects: A Hypothesis with Growth Hormone. Ocul Surf 2015 7;13(3):204–212.e1. doi: 10.1016/j.jtos.2014.12.005 26045234 10.1016/j.jtos.2014.12.005 PMC4498999

[4] Ljubimov AV, Saghizadeh M. Progress in corneal wound healing. Prog Retin Eye Res. 2015 11;49:17–45. doi: 10.1016/j.preteyeres.2015.07.002 26197361 10.1016/j.preteyeres.2015.07.002 PMC4651844

[5] Liu CY, Kao WW. Corneal Epithelial Wound Healing. Prog Mol Biol Transl Sci 2015;134:61–71. doi: 10.1016/bs.pmbts.2015.05.002 26310149 10.1016/bs.pmbts.2015.05.002

[6] Cao Z, Saravanan C, Chen WS, Panjwani N. Examination of the role of galectins in cell migration and re-epithelialization of wounds. Methods Mol Biol 2015;1207:317–326. doi: 10.1007/978-1-4939-1396-1_21 25253150 10.1007/978-1-4939-1396-1_21

[7] Oudhoff MJ, Bolscher JG, Nazmi K, Kalay H, van 't Hof W, Amerongen AV, et al Histatins are the major wound-closure stimulating factors in human saliva as identified in a cell culture assay. FASEB J. 2008 11;22(11):3805–12. doi: 10.1096/fj.08-112003 18650243 10.1096/fj.08-112003

[8] Melino S, Santone C, Di Nardo P, Sarkar B. Histatins: salivary peptides with copper(II)- and zinc(II)-binding motifs: perspectives for biomedical applications. FEBS J 2014 2;281(3):657–672. doi: 10.1111/febs.12612 24219363 10.1111/febs.12612

[9] Gipson IK, Spurr-Michaud S, Argüeso P, Tisdale A, Ng TF, Russo CL. Mucin gene expression in immortalized human corneal–limbal and conjunctival epithelial cell lines. Investigative ophthalmology & visual science. 2003 6 1;44(6):2496–506. 12766048 10.1167/iovs.02-0851

[10] Abdel-Naby W, Cole B, Liu A, Liu J, Wan P, Guaiquil VH, Schreiner R, Infanger D, Lawrence BD, Rosenblatt MI. Silk-Derived Protein Enhances Corneal Epithelial Migration, Adhesion, and ProliferationSilk Derived Protein Enhances Corneal Epithelial Growth. Investigative Ophthalmology & Visual Science. 2017 3 1;58(3):1425–33. 28257533 10.1167/iovs.16-19957 PMC6022413

[11] Van Goethem F, Adriaens E, Alepee N, Straube F, De Wever B, Cappadoro M, et al Prevalidation of a new in vitro reconstituted human cornea model to assess the eye irritating potential of chemicals. Toxicol In Vitro 2006 2;20(1):1–17. doi: 10.1016/j.tiv.2005.05.002 16019187 10.1016/j.tiv.2005.05.002

[12] Ashby B, Garrett Q, Willcox M. Corneal Injuries and Wound Healing–Review of Processes and Therapies. Austin J Clin Ophthalmol 2014 3 21;1(4):1017.

[13] Borrelli M, Schröder C, Dart JK, Collin JR, Sieg P, Cree IA, et al Long-term follow-up after submandibular gland transplantation in severe dry eyes secondary to cicatrizing conjunctivitis. Am J Ophthalmol. 2010 12;150(6):894–904. doi: 10.1016/j.ajo.2010.05.010 20920813 10.1016/j.ajo.2010.05.010

[14] van ‘t Hof W, Oudhoff MJ, Veerman ECI. Antimicrobial peptides and innate immunity Hiemstra PS, Zaat SAJ, editors. Histatins: Multifunctional Salivary Antimicrobial Peptides. 1st ed. Basel: Springer; 2013 p. 167–182.

[15] Shah D, Ali M, Pasha Z, Jaboori AJ, Jassim SH, Jain S, et al Histatin-1 is a marker for human lacrimal epithelium. Plos One. 2016 1 29;11(1):e0148018 doi: 10.1371/journal.pone.0148018 26824896 10.1371/journal.pone.0148018 PMC4732786

[16] Baum BJ, Bird JL, Millar DB, Longton RW. Studies on histidine-rich polypeptides from human parotid saliva. Arch Biochem Biophys 1976 12;177(2):427–436. 1015827 10.1016/0003-9861(76)90455-0

[17] Ali M, Shah D, Pasha Z, Jassim SH, Jaboori AJ, Setabutr P, et al Evaluation of Accessory Lacrimal Gland in Muller's Muscle Conjunctival Resection Specimens for Precursor Cell Markers and Biological Markers of Dry Eye Disease. Curr Eye Res 2016 9 9:1–7. 10.1080/02713683.2016.1214966 PMC5551074 27612554

[18] Helmerhorst EJ, van't Hof W, Breeuwer P, Veerman EC, Abee T, Troxler RF, et al Characterization of histatin 5 with respect to amphipathicity, hydrophobicity, and effects on cell and mitochondrial membrane integrity excludes a candidacidal mechanism of pore formation. J Biol Chem. 2001 2 23;276(8):5643–9. doi: 10.1074/jbc.M008229200 11099499 10.1074/jbc.M008229200

[19] Helmerhorst EJ., Troxler RF, Oppenheim FG. The human salivary peptide histatin 5 exerts its antifungal activity through the formation of reactive oxygen species. Proc Natl Acad Sci U S A. 2001 12 4; 98(25): 14637–14642. doi: 10.1073/pnas.141366998 11717389 10.1073/pnas.141366998 PMC64734

[20] Oudhoff MJ, Kroeze KL, Nazmi K, van den Keijbus PA, van 't Hof W, Fernandez-Borja M, et al Structure-activity analysis of histatin, a potent wound healing peptide from human saliva: cyclization of histatin potentiates molar activity 1,000-fold. FASEB J 2009 11;23(11):3928–3935. doi: 10.1096/fj.09-137588 19652025 10.1096/fj.09-137588

[21] van Dijk IA, Nazmi K, Bolscher JG, Veerman EC, Stap J. Histatin-1, a histidine-rich peptide in human saliva, promotes cell-substrate and cell-cell adhesion. The FASEB Journal. 2015 8 1;29(8):3124–32. doi: 10.1096/fj.14-266825 25903106 10.1096/fj.14-266825

[22] Campese M, Sun X, Bosch JA, Oppenheim FG, Helmerhorst EJ. Concentration and fate of histatins and acidic proline-rich proteins in the oral environment. Archives of oral biology. 2009 4 30;54(4):345–53 doi: 10.1016/j.archoralbio.2008.11.010 19159863 10.1016/j.archoralbio.2008.11.010 PMC2680473

[23] Kepp O, Galluzzi L, Lipinski M, Yuan J, Kroemer G. Cell death assays for drug discovery. Nature reviews Drug discovery. 2011 3 1;10(3):221–37. doi: 10.1038/nrd3373 21358741 10.1038/nrd3373

[24] Fotakis G, Timbrell JA. In vitro cytotoxicity assays: comparison of LDH, neutral red, MTT and protein assay in hepatoma cell lines following exposure to cadmium chloride. Toxicology letters. 2006 1 5;160(2):171–7. doi: 10.1016/j.toxlet.2005.07.001 16111842 10.1016/j.toxlet.2005.07.001

[25] Riss TL, Moravec RA, Niles AL, Duellman S, Benink HA, Worzella TJ, et al. Cell Viability Assays. In: Sittampalam GS, Coussens NP, Brimacombe K, Grossman A, Arkin M, Auld D, et al, editors. Assay Guidance Manual Bethesda (MD); 2004.

[26] Duque A, Rakic P. Different effects of bromodeoxyuridine and [3H] thymidine incorporation into DNA on cell proliferation, position, and fate. Journal of Neuroscience. 2011 10 19;31(42):15205–17. doi: 10.1523/JNEUROSCI.3092-11.2011 22016554 10.1523/JNEUROSCI.3092-11.2011 PMC3225276

[27] Wang L, Ko CY, Meyers EE, Pedroja BS, Pelaez N, Bernstein AM. Concentration-dependent effects of transforming growth factor β1 on corneal wound healing. Mol Vis. 2011;17:2835–46. 22128231 PMC3224828

